# The Predictive Value of Monocytes in Immune Microenvironment and Prognosis of Glioma Patients Based on Machine Learning

**DOI:** 10.3389/fimmu.2021.656541

**Published:** 2021-04-16

**Authors:** Nan Zhang, Ziyu Dai, Wantao Wu, Zeyu Wang, Hui Cao, Yakun Zhang, Zhanchao Wang, Hao Zhang, Quan Cheng

**Affiliations:** ^1^ Department of Neurosurgery, Xiangya Hospital, Central South University, Changsha, China; ^2^ College of Bioinformatics Science and Technology, Harbin Medical University, Harbin, China; ^3^ Department of Oncology, Xiangya Hospital, Central South University, Changsha, China; ^4^ National Clinical Research Center for Geriatric Disorders, Xiangya Hospital, Central South University, Changsha, China; ^5^ Department of Psychiatry, The Second People’s Hospital of Hunan Province, The Hospital of Hunan University of Chinese Medicine, Changsha, China; ^6^ Department of Orthopaedics, Changzheng Hospital, Naval Medical University, Shanghai, China

**Keywords:** monocyte, glioma microenvironment, immune infiltration, machine learning, immunotherapy, prognostic model

## Abstract

Gliomas are primary malignant brain tumors. Monocytes have been proved to actively participate in tumor growth. Weighted gene co-expression network analysis was used to identify meaningful monocyte-related genes for clustering. Neural network and SVM were applied for validating clustering results. Somatic mutation and copy number variation were used for defining the features of identified clusters. Differentially expressed genes (DEGs) between the stratified groups after performing elastic regression and principal component analyses were used for the construction of risk scores. Monocytes were associated with glioma patients’ survival and exhibited high predictive value. The prognostic value of risk score in glioma was validated by the abundant expression of immune checkpoint and metabolic profile. Additionally, high risk score was positively associated with the expression of immunogenic and antigen presenting factors, which indicated high immune infiltration. A prognostic model based on risk score demonstrated high accuracy rate of receiver operating characteristic curves. Compared with previous studies, our research dissected functional roles of monocytes from large-scale analysis. Findings of our analyses strongly support an immune modulatory and prognostic role of monocytes in glioma progression. Notably, monocyte could be an effective predictor for therapy responses of glioma patients.

## Introduction

Gliomas are one of the most malignant solid cancer types, which grade 2 and grade 3 glioma are defined as diffuse lower-grade glioma (LGG) and grade 4 glioma is defined as glioblastoma (GBM) based on the WHO 2016 classification (1). GBM, with the highest incidence rate (3.23 per 100,000 population) in United states, accounted for the majority of gliomas (57.7%) (2). The 10-year survival rate of LGG is 47% whereas the median overall survival (OS) time of GBM is less than 3 years (3). Recently, increasing molecular markers have been identified for prediction of glioma patient survival rate, including mutational status, and DNA methylation (4, 5). Given that, WHO proposed an updated grading system for CNS tumors integrating molecular diagnosis (6). However, the inevitable tumor recurrence and drug resistance due to the high heterogeneity of gliomas make it still urgent to identify novel biomarkers to help illustrate the pathological mechanism of gliomas and develop the corresponding therapeutic strategies.

Tumor microenvironment (TME), consisting of noncancerous cells and tumor associated biomolecules, have become increasingly attractive as potential targets for the treatment of gliomas (7). Accumulating evidence has demonstrated the immunosuppressive context in TME of gliomas, such as tumor associated macrophages (TAMs), regulatory T cells (Tregs), cancer associated fibroblasts (CAFs), myeloid derived suppressor cells (MDSCs), and monocytes (7, 8). Monocytes, emerged as important regulators of cancer progression, are innate immune cells of the mononuclear phagocyte system. Monocytes perform diverse functions that contribute to both pro- and antitumoral immunity during cancer development, including phagocytosis, secreting tumoricidal mediators, promoting of angiogenesis, remodeling extracellular matrix, and recruiting lymphocytes (8). Monocytes comprise as many as 30–50% of all cells in GBM microenvironment (9). Previous study has proved that monocytes closely adhere to GBM via vascular cell adhesion molecule-1 (*VCAM-1*) (10). Notably, monocytes also serve as the important source of TAMs and dendritic cells (DCs) that shape a more permissive TME (11). Moreover, monocyte-mediated nano drug delivery in GBM has been proposed and proved with effective cancer cell damage. Although several studies have highlighted the potential roles of monocytes in tumor growth, the in-depth mechanism of monocytes in TME and its overall prognostic value in gliomas has not been fully elucidated due to its eventual destiny of differentiation.

Weighted gene co-expression network analysis (WGCNA) has been known for its ability to explore the specific genes related to clinical traits. In this study, WGCNA was employed to identify meaningful monocyte-related gene modules in glioma patients. Genes within the identified module were extracted for clustering. Machine learning including neural network and Support Vector Machines (SVM) was used to validate the clustering results. Significant differentially expressed genes (DEGs) between the stratified groups after performing elastic regression and Principal component analyses (PCA) were used for the construction of risk scores. Risk scores could also predict immunotherapeutic efficiency. These results are expected to promote the development of novel therapeutic targets based on monocytes and provide the basis for future research on monocytes in gliomas. Besides, given the current shortcoming in diagnostic and therapeutic options in GBM, the remarkable prognostic value of monocytes can better achieve precise medicine and promote the clinical management of GBM patients.

## Methods

### Patient and Cohort Inclusion

2405 diffuse glioma samples were collected from three databases: The Cancer Genome Atlas (TCGA), Chinese Glioma Genome Atlas (CGGA), and Gene Expression Omnibus (GEO). For the TCGA cohort (672 glioma samples), the RNA-seq data and corresponding clinical information were retrieved from TCGA database (http://cancergenome.nih.gov/). Three CGGA validation cohorts were employed in this study, including two RNA-seq cohorts (CGGA325 and CGGA693) and a microarray cohort (CGGAarray). The RNA-seq and microarray data, clinical and survival information were downloaded from the CGGA database (http://www.cgga.org.cn). Expression matrices of GSE108474 (414 glioma samples) were obtained from the GEO database (https://www.ncbi.nlm.nih.gov/geo/).

### WGCNA Identifying Monocytes Related Genes

The WGCNA package in R version 3.6.1 was used to perform WGCNA. The association between individual genes and monocyte densities was quantified by gene significance, and the correlation between module eigengenes and gene expression profiles was represented by module membership. A power of β = 2 and a scale-free R2 = 0.89 were set as soft-threshold parameters to ensure a scale-free topology network. A total of seven modules were generated, and turquoise module showing the strongest correlation was used for further analysis. Genes within the turquoise module were thus chosen for GO (gene ontology) and KEGG (Kyoto Encyclopaedia of Genes and Genomes) functional enrichment analyses. Metascape (https://metascape.org/) was also used for functional annotation of turquoise module genes.

### Delineation and Validation of Immune Subtypes

Based on the 806 genes extracted from turquoise module, we applied consensus clustering algorithm of partition around medoids (PAM) to identify robust clusters of TCGA patients (12). The cumulative distribution function (CDF) and consensus heatmap were used to assess the optimal *K* value of 2. To validate the immune subtypes in three CGGA cohorts, we trained a neural network classifier in the discovery cohort to predict the immune subtypes for patients in the validation cohort based on 300 overlapped module-derived genes in TCGA and three CGGA cohorts using R package Rcpp, RSNNS, and “e1071”. Among the three learning functions (Quickprop, BackpropBatch, SCG), Quickprop was used for the training. The clustering results were further validated by SVM using R package caret and “e1071”. Three types of models (C-classification, nu-classification, one-classification) and four types of kernels (linear, polynomial, radial, sigmoid) in SVM were analyzed. The combination of C-classification and radial was found with the highest accuracy.

### Genomic Alterations in Immune Subtypes

Somatic mutations and somatic copy number alternations (CNAs) which corresponded to the cases with RNA-seq data, were downloaded from the TCGA database. GISTIC analysis was performed to determine the genomic event enrichment. CNAs associated with the two clusters and the threshold copy number at alteration peaks were obtained using GISTIC 2.0 analysis (https://gatk.broadinstitute.org).

### Annotation of the Immune Infiltrating Microenvironment

ESTIMATE was performed to evaluate the immune cell infiltration level (immune scores) and stromal content (stromal scores) for each sample. The enrichment levels of 64 immune signatures were quantified by the xCell algorithm (13). The relative fraction of 22 immune cell types in tumor tissues were estimated using CIBERSORT algorithm (14). Gene set variation analysis (GSVA) was performed to study GO pathways, and GO items with p value < 0.05 were identified. Seven types of classified immune checkpoints signaling pathways were investigated from two previous published studies (15, 16).

### Identification of an Immune-Related Signature

Univariate Cox regression analysis was performed to determine the differentially expressed immune genes with prognostic significance with a p value < 0.05 between subtypes. Elastic regression analysis and PCA were further used to calculate the risk scores of patients. The extracted principal component 1 served as the signature score. The risk score of each patient after the prognostic value of gene signature score was obtained by the following calculation: ΣPC1i - ΣPC1j, where i represented the expression of genes with HR>1, and j the expression of genes with HR<1.

### Prediction of Immunotherapy Response

The IMvigor210 cohort, which is an urothelial carcinoma cohort treated with the anti‐*PD‐L1* antibody atezolizumab was used for prediction of patient response to immunotherapy (16). Based on the Creative Commons 3.0 License, complete expression data and clinical data were downloaded from http://research-pub.Gene.com/IMvigor210CoreBiologies. Raw data were then normalized using the DEseq2 R package, and the count value was transformed into the TPM value.

### Construction and Validation of a Prognostic Model

Ultimately, nomogram is a form of visualized multi-factor regression analysis commonly used for cancer survival rate prediction. Variables selected for construction of the nomogram included the calculated prognostic scores, ages, pathological stages of glioma and mutation status. Univariate and multivariate regression analyses were also used to evaluate the prognostic value of these factors.

### Statistical Analysis

Kaplan-Meier curves with log-rank test were used to assess survival difference between groups. The univariate and multivariate Cox regression analyses were performed to detect the prognostic factors. Pearson correlation and distance correlation analyses were used to calculate correlation coefficients. Contingency tables were analyzed by χ^2^ contingency test. The OS and risk scores were calculated using the R package survival and cutoff values determined. Based on the dichotomized risk scores, patients were grouped as with high or low risk score in each data set, and the computational batch effect was reduced by the R package sva. Data were visualized using the R package ggplot2. OncoPrint was used to delineate the mutation landscape of TCGA by the maftools R package (17). All survivorship curves were generated using R package survminer. Heatmaps were generated based on pheatmap. All statistical analyses were conducted using R software. P < 0.05 was considered statistically significant.

## Results

### Identification of Monocyte Density as a Potential Prognostic Marker

**Figure 1:**
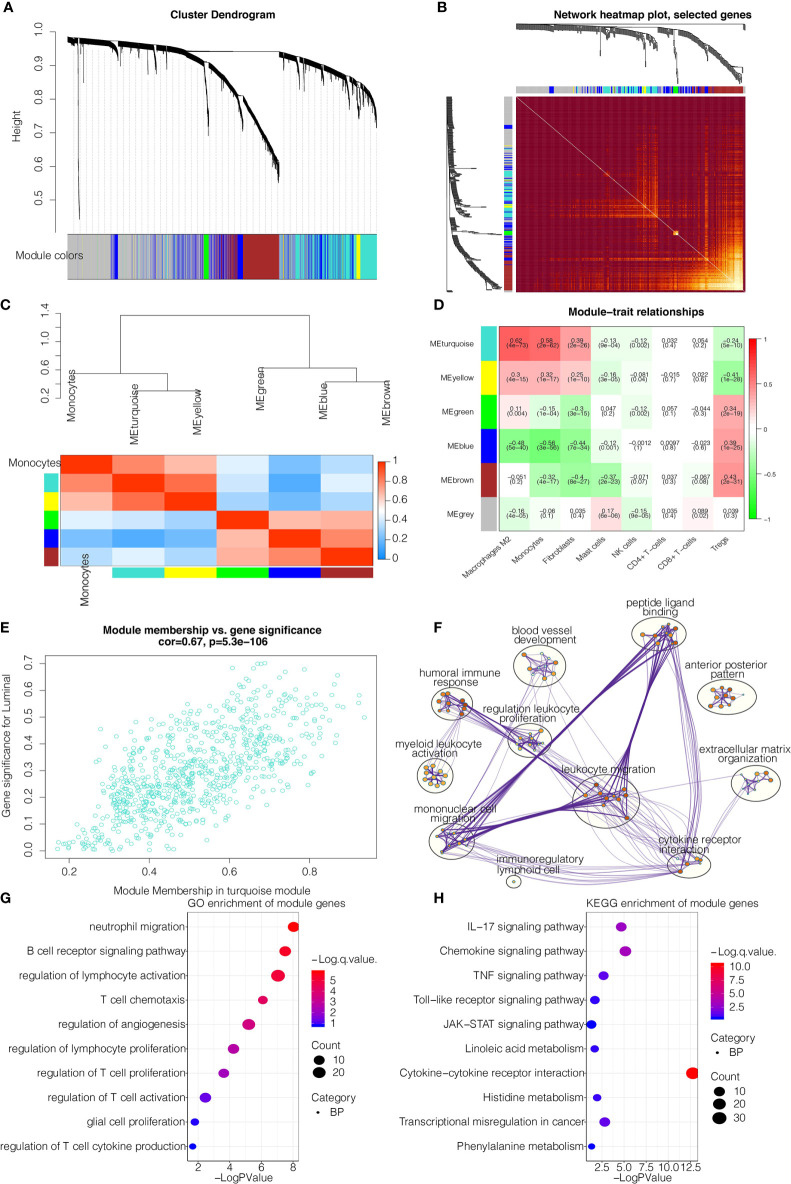
WGCNA for the monocyte-related genes. **(A)** Cluster dendrogram generating gene modules. **(B)** Tomplot depicting the random 400 genes. **(C)** Hierarchical clustering dendrogram of module. **(D)** Correlation analysis of modules and cell types. **(E)** Scatterplot demonstrating the correlation of intramodular connectivity and monocytes. **(F)** Metascape for the functional annotation of key genes in module turquoise. **(G)** GO functional enrichment analysis of key genes in module turquoise. **(H)** KEGG functional enrichment analysis of key genes in module turquoise.

The flow chart of our study design was shown in **Figure S1A**. We sought to determine the prognostic value of monocytes in glioma by studying the monocyte-related genes using WGCNA. After stratifying patients by high and low median levels of monocytes, survival analysis revealed a clear distinction between the two subtypes in LGG, GBM, and pan-gliomas from TCGA, respectively (**Figure S1B**). The expression level of monocyte could also stratify patients in CGGAarray, CGGA325, and CGGA693, respectively (**Figure S1C**). To evaluate the potential prognostic value of monocytes, we performed WGCNA for monocyte-specific genes. A power β=2 was selected as the software threshold for a scale-free network construction. Seven modules were identified by clustering dendrogram (**Figure 1A**). Tomplot depicting the random 400 genes within the clustering dendrogram (**Figure 1B**). The correlation between the turquoise module and xCell-defined monocytes was 0.58, indicating a selective expression of the turquoise module in monocytes (**Figures 1C, D**). Once established the turquoise module as the one with the highest significance, we investigated the correlation between the intramodular connectivity and monocytes, which reached 0.67 (**Figure 1E**). Metascape revealed that turquoise-derived genes were enriched in leukocyte migration and mononuclear cell migration (**Figure 1F**). GO functional enrichment analysis found that the genes were concentrated in pathways involving neutrophil migration and regulation of lymphocyte activation (**Figure 1G**). KEGG analysis showed that the genes were enriched in the cytokine-receptor interaction (**Figure 1H**).

**Figure 2:**
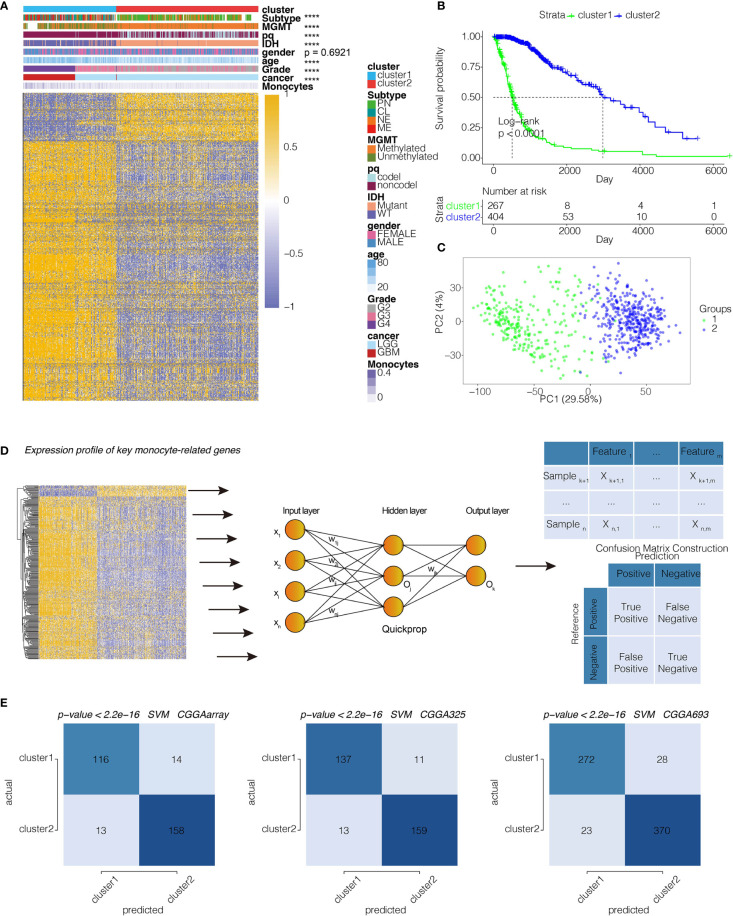
Machine learning for validation of clustering based on monocyte-related genes. **(A)** Clustering dendrogram demonstrating good separation of the two clusters by traits. ****P < 0.0001. **(B)** Kaplan-Meier survival analysis of the two clusters. **(C)** Sample clustering by PCA in the TCGA dataset. **(D)** Construction of clustering in CGGAarray, CGGA325, and CGGA693 based on the clustering in TCGA by neural network learning function of Quickprop. Schematic diagram of the neural network. **(E)** Validation of clustering by SVM algorithm in CGGAarray, CGGA325, and CGGA693. Contingency tables showing the high consistency between predicted clusters from SVM and actual clusters from neural network.

We subsequently extracted 806 genes from module turquoise by WGCNA. PAM was performed for glioma patients with the corresponding gene expression profiles in TCGA cohort (**Figure 2A**). The optimal number of clusters was evaluated by ConsensusClusterPlus package (**Figure S2A**). Clustering results were most stable when the number was set to two (*K*=2). The delineated groups based on the 806 genes showed distinct patterns of clinical traits and monocyte levels with statistical significance (**Figure 2A**). Survival analyses of the two clusters confirmed an obviously lower survival probability curve for cluster 1 (**Figure 2B**). PCA managed to differentiate the samples from the TCGA dataset (**Figure 2C**). Subsequently, combining the gene expression profiles from three CGGA cohorts, 300 genes were identified from these 806 genes by neural network to validate the clustering results (**Figure 2D**). Samples were then clustered into two groups with high or low death risk by pamr in three CGGA cohorts, respectively (**Figures S2B-D**). SVM was performed for validation of the clustering as well, which the contingency table showed the consistency in clustering results among SVM and neural network (**Figure 2E**). Survival analyses of the two clusters confirmed an obviously lower survival probability curve for cluster 1 (**Figures S2E–G**). PCA also managed to differentiate the samples from three individual datasets (**Figures S2H–J**).

### Clinical traits and TME Characteristics of the Monocyte-Stratified Groups

**Figure 3:**
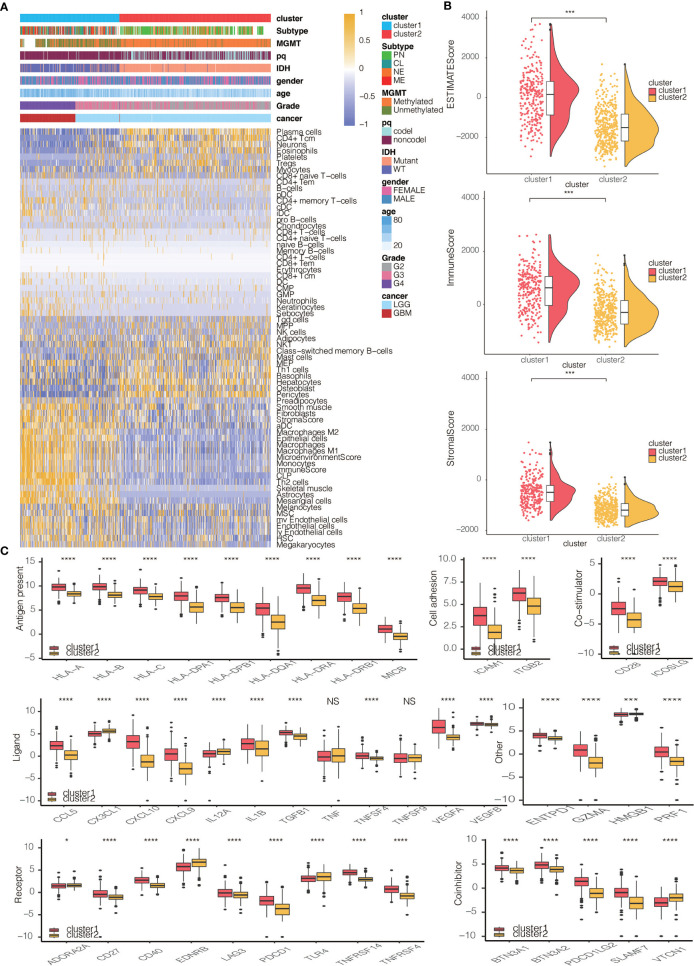
Immune characteristics of the two clusters. **(A)** Dendrogram correlating the levels of 64 cell types calculated by xCell and clusters in TCGA. **(B)** ESTIMATEScores, ImmuneScores and StromalScores of the two clusters in TCGA. **(C)** Molecule levels of seven types of immune checkpoints in two clusters in TCGA. *P < 0.05, ***P < 0.001, ****P < 0.0001. NS, not statistically significant.

We then proceeded to investigate the TME characteristics of the two clusters. The expression difference of the levels of 64 cell types in two defined subtypes were investigated in TCGA and three CGGA cohorts (**Figures 3A** and **S3A**). It was found that increased cells such as fibroblasts, DCs, M2 macrophages and monocytes were related to cluster 1 with worse survival probability. Moreover, CIBERSORT algorithm showed that the expression of several types of immune cells including M0/1/2 macrophages, DCs, and neutrophils were higher in cluster 1 in TCGA, CGGAarray, CGGA325, and CGGA693, respectively (**Figures S3B**, **S4A**, **S5A**, **S6A**). The association between ESTIMATE scores of the immune infiltrating microenvironment, an indicator of the cancer biological behaviour, and clusters, as well as levels of immune cells was examined in TCGA and three CGGA cohorts (**Figures 3B** and **S4B**, **S5B**, **S6B**). ESTIMATEScores, ImuneScores and StromalScores were all higher in cluster 1 than in cluster 2 (**Figures 3B** and **S4B**, **S5B**, **S6B**). We then compared the levels of several series of immune checkpoint molecules related to antigen presentation, cell surface receptor, coinhibition, ligand and cell adhesion between the two clusters. Immune checkpoint markers tended to be overexpressed in cluster 1 (**Figures 3C** and **S4C**, **S5C**, **S6C**).

The pathological gradings of glioma were also significantly different between clusters 1 and 2 (p<2.2e-16), with a higher gradings in cluster 1 in TCGA and three CGGA cohorts (**Figure S7A**). The proportions of samples with *IDH* wildtype *(*WT) and chromosome 1p/19q codeletion in cluster 1 were higher than those in cluster 2 (**Figures S7B, S7C**), also indicating a more malignant propensity in cluster 1. Results regarding the proportion of patients with *MGMT* promoter methylation were less universal, with data from the TCGA database showing the most significant difference while data from the other three databases statistically insignificant difference (**Figure S7D**). The proportions of the four GBM subtypes in clusters 1 and 2 were significantly different in TCGA (p<2.2e-16), showing that the more malignant CL and ME subtypes accounted for the majority of cluster 1 samples (**Figure S7E**).

The expression differences of hypoxia pathways in two clusters were explored using GSVA. Investigated pathways included cell response regulation, hypoxia-induced intrinsic apoptosis, Hypoxia-Inducible Factor 1α (HIF1A) and others. These pathways were found to be more activated in cluster 1 in TCGA and three CGGA cohorts, suggesting a tendency for cell hypoxia, which is a universal marker for malignant tumor proliferation, in this group (**Figures S8A–D**). We also interrogated the relationship between metabolic pathways, such as pyrimidine synthesis and sulfur metabolism, and subtypes. The metabolic pathways were overrepresented in cluster 1, proving a more active proliferation of glioma cells in these samples (**Figures S8A–D**).

### Monocyte-Enriched Group Showed More Malignant Genomic Features

**Figure 4:**
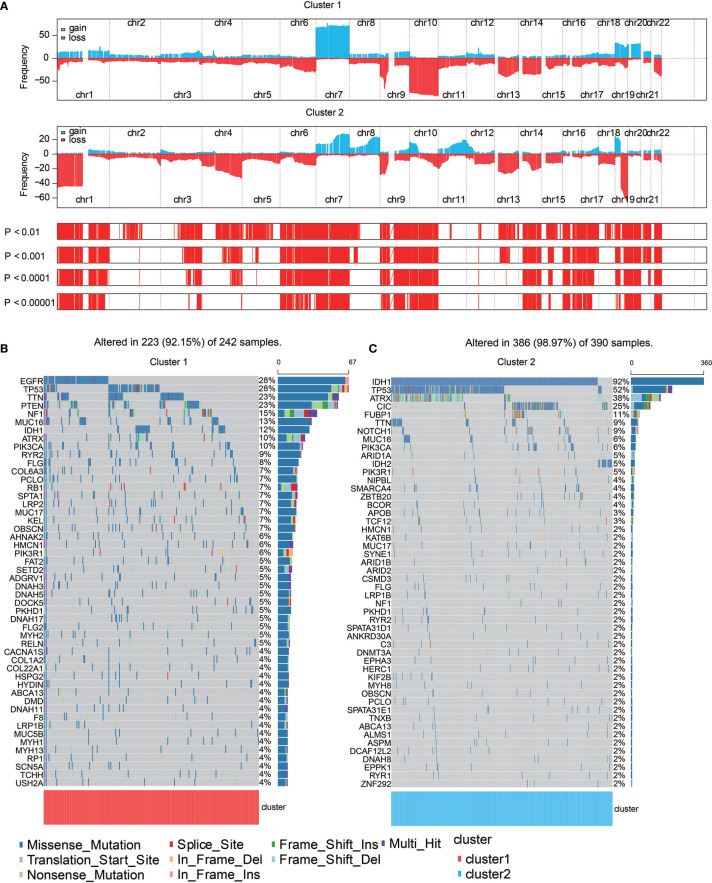
Genomic features of the two clusters. **(A)** Distribution of gain or loss of function mutation in the 22 human chromosomes in the two clusters. Amplification of genes is marked in blue. Deletion of genes is marked in red. **(B)** List of the most frequently altered genes in clusters 1. **(C)** List of the most frequently altered genes in clusters 2. Nine mutation types were exhibited.

Somatic mutation analysis and copy number variation (CNV) were performed using the TCGA dataset to explore genomic traits of the two clusters (**Table S1**). A global CNV profile was obtained by comparing the two clusters (**Figure 4A** and **Table S2**). According to somatic mutation analysis, mutations in *EGFR* (28%), *TP53* (28%), *PTEN* (23%) and *TTN* (23%) were most highly enriched in cluster 1 (**Figure 4B**). In comparison, *IDH1* (92%), *TP53* (52%), *ARTX* (38%) and *CIC* (25%) mutations were enriched in cluster 2 (**Figure 4C**). Missense mutation was the predominant gene alteration type in all these genes except for *ATRX*, in which frame-shifting deletion was the most common type.

**Figure 5:**
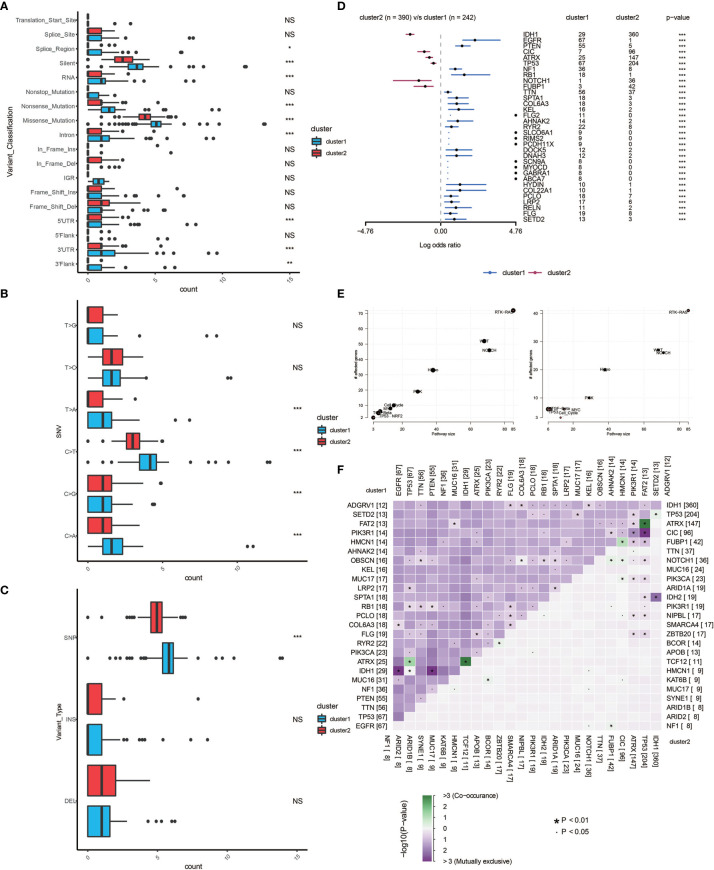
Genomic alterations in the two clusters. Frequency comparison according to types of mutation **(A)**, SNV **(B)**, INDEL and SNV **(C)** between the two clusters. **(D)** The Forest plot listing the top 17 most mutated genes between the two clusters. **(E)** Demonstration of the pathways involved in cancer biology in the two clusters. **(F)** The heatmap showing the concurrence or mutual exclusivity of the top 25 most mutated genes in the two clusters. *p < 0.05, *p < 0.01, ***P < 0.001, ****P < 0.0001. NS, not statistically significant.

Different types of somatic mutations, including the single-nucleotide variant (SNV), single-nucleotide polymorphism (SNP), insertion, deletion and intergenic region (IGR), were analyzed using the R package. Silent, nonsense, missense, intronic, 5’ and 3’ UTR mutations were more common in cluster 1 than in cluster 2 (**Figure 5A**). Among the detected SNVs, C>T appeared to be the most common mutation in cluster 1 (**Figure 5B**). The T to A, C to T and C to A mutations occurred more frequently in cluster 1 than in cluster 2. While the frequencies of insertion and deletion were not statistically different between the two clusters, SNPs were significantly more common in cluster 1 (**Figure 5C**). The top 33 most mutated cancer-related genes were listed in **Figure 5D**. Common carcinogenic pathways were more active in cluster 1 (**Figure 5E**). The strongest co-occurrent pairs of gene alteration in cluster 1 were *ATRX*-*TP53* and *ATRX*-*IDH1*, which was in accordance with previous reports (18–20). It was suggested that acquisition of a second cancer-related gene alteration may dictate the development of certain tumor types, and that *TP53*, *IDH1*, *ATRX* are functionally linked (**Figure 5F**) (20, 21 *).* On the other hand, the most mutually exclusive pairs were *PTEN*-*IDH1* and *EGFR*-*IDH1* (**Figure 5F**).

### Generation of Risk Score and Its Functional Annotation

**Figure 6:**
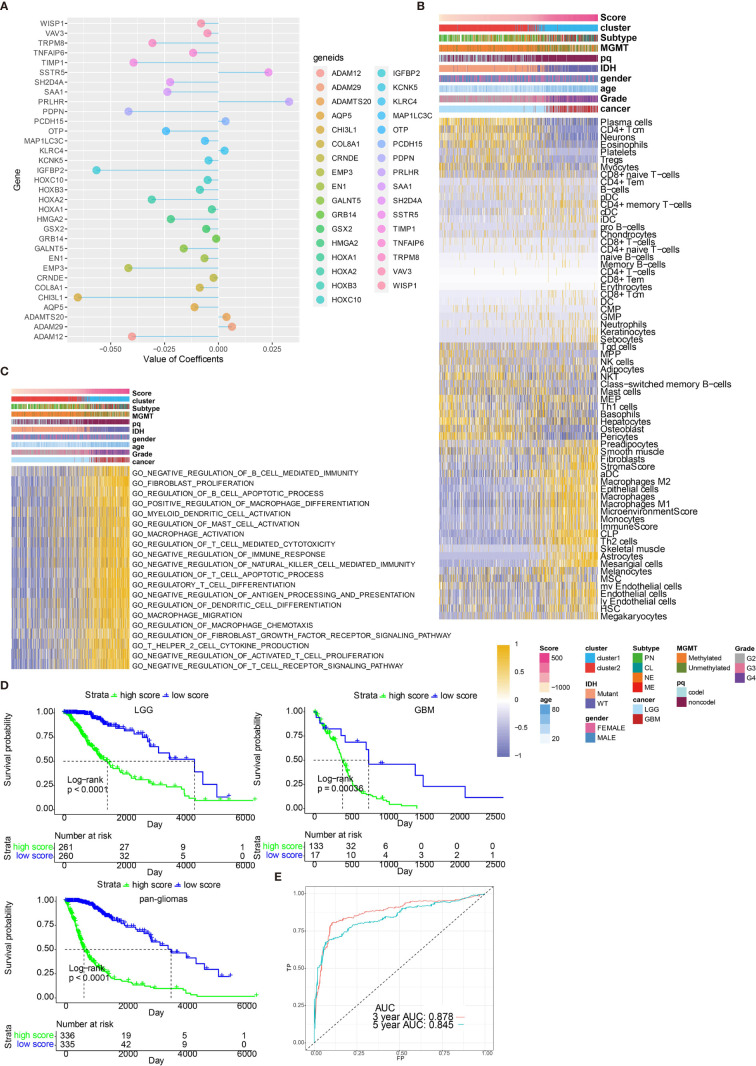
Functional annotation of risk scores. **(A)** Elastic net regression analysis and PCA obtained 33 monocyte-related genes and their coefficients. **(B)** Dendrogram correlating the risk score and 64 cell types. **(C)** GO functional enrichment analysis correlating different immune regulatory processes with risk score. **(D)** Survival analyses of risk scores in pan-glioma, LGG and GBM groups from TCGA. D, Hazard ratios of risk scores in different cancer types. **(E)** ROC curve measuring the sensitivity of risk score in predicting patient’s 3 years and 5 years survival status. The area under the ROC curve was 0.878 and 0.845, respectively.

By performing elastic net regression analysis and PCA algorithm (**Figure S9A**), 33 monocyte-related genes were derived from the 300 genes and their coefficients were obtained (**Figure 6A**). The monocyte-related gene signature was used to calculate risk scores by PCA. Sankey plot revealed a high consistency between monocyte-related clusters and risk scores (**Figure S9B**). The correlation of the expression levels of 64 cell types and risk scores was then evaluated. There was a positive correlation between the scores and the levels of fibroblasts, M2 macrophages, DCs, and monocytes (**Figure 6B**). Pathways related to macrophage activation and migration, dendritic cell differentiation and negative regulation of T cell proliferation were more active in the samples with higher scores (**Figure 6C**). In the TGCA dataset, survival analysis demonstrated a good separation of patients with different death risks by high and low risk scores (**Figure 6D**). The prognostic value of risk scores was further validated in CGGAarray, CGGA325, CGGA693, and GSE108474 datasets (**Figure S9C**). The receiver operating characteristic (ROC) analyses with the Area Under the Curve (AUC) of 0.878 and 0.845 confirmed that risk score was a prognostic biomarker in predicting 3 years and 5 years survival status of glioma patients (**Figure 6E**).

### Construction of a Prognostic Nomogram Based on Risk Scores

After establishing monocyte density as a suitable marker for survival prediction of gliomas, we further investigated its prediction efficiency by developing a prognostic nomogram. Combing prognostic factors, including risk scores, patient ages, tumor grades, *IDH* mutation, and chromosome 1p/19q codeletion, a prognostic nomogram was developed (**Figure S10A**). In TCGA dataset, the Kaplan-Meier survival curve demonstrated a good discrimination of survival probabilities of the two clusters (p<0.0001) (**Figure S10B**). The ROC curve confirmed the discriminative ability of this nomogram (AUC=0.802, **Figure S10C**). Predicted probabilities corresponded well with the actual one- to five-year overall survival rates of glioma patients (**Figure S10D**). The efficiency of the prognostic model was validated in CGGA 693 cohort. The Kaplan-Meier survival curve demonstrated a good discrimination of survival probabilities of the two clusters (p<0.0001) (**Figure S10E**). The ROC curve confirmed the discriminative ability of this nomogram (AUC=0.737, **Figure S10F**). Predicted probabilities corresponded well with the actual four-year overall survival rates of glioma patients (**Figure S10G**).

### Monocyte-Stratified Groups Predicted Response to Immunotherapies

**Figure 7:**
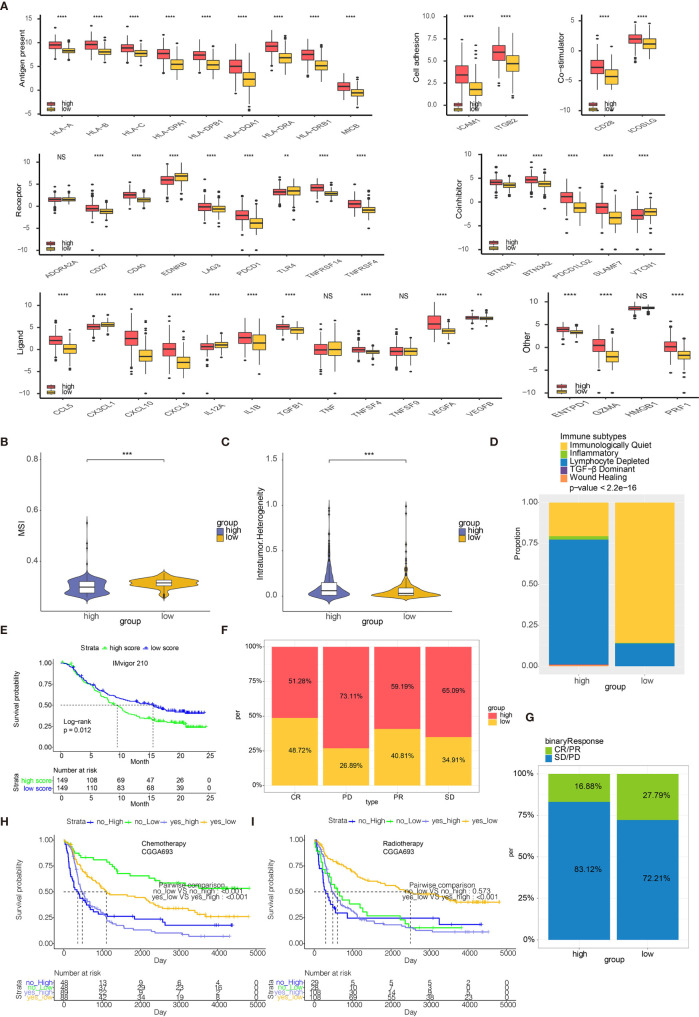
Risk scores predict immunotherapy response. **(A)** Molecule levels of immune checkpoints in two risk score groups in TCGA. **(B)** MSI in high and low risk score. **(C)** Intratumor Heterogeneity in high and low risk score. **(D)** Distribution of six immune subtypes in two risk score groups in TCGA. **(E)** Kaplan-Meier curve of high and low risk score groups in IMvigor210 cohort. **(F)** The bar chart showing proportions of high and low risk scores. **(G)** The bar chart showing proportions of CR/PR and SD/PD patients in high and low risk score groups. **(H)** Kaplan-Meier curve of high and low risk score groups in patients receiving chemotherapy from CGGA693. **(I)** Kaplan-Meier curve of high and low risk score groups in patients receiving radiotherapy from CGGA693. **P < 0.01, ***P < 0.001, ****P < 0.0001. NS, not statistically significant.

High risk scores were associated with several immune checkpoint molecules including PDCD1, PDCD1LG2, and LAG3 (**Figure 7A**). Except for immune checkpoint molecules, the intrinsic immune escape mechanism was reported to include tumor immunogenicity and antigen presentation capacity (22). Factors associated with tumor immunogenicity was first assessed in glioma samples from TCGA (23). High risk score group exhibited lower microsatellite instability (MSI) and higher level of intratumor heterogeneity (**Figures 7B, C**, respectively). High risk score group presented higher silent mutation rate, number of segements, homologous recombination deficiency (HRD), aneuploidy score, and fraction altered that were all crucial indicators for genomic alterations (**Figures S11A–E**). Cancer testis antigen (CTA) and neoantigens were vital sources of tumor-specific antigens, and they were both higher in high risk score group (**Figures S11F, S11G**). Further, high risk score group exhibited higher level of macrophage regulation, lymphocyte infiltration signature score, leukocyte fraction, TCR Shannon, and TCR richness, all of which were significant indicators for antigen presentation capacity (**Figures S11H–L**). Six immune subtypes including Wound Healing, IFN-γ Dominant, Inflammatory, Lymphocyte Depleted, Immunologically Quiet, and tumor growth factor-β (TGF-β) Dominant have been previously identified across cancer types (23). Lymphocyte Depleted, representing an immune cold microenvironment, was more frequently observed in high risk score group (**Figure 7D**). We evaluated whether risk scores were able to predict therapeutic effects of immune blockade treatment. High and low risk scores succeed in stratifying patients by survival probability from the IMvigor210 cohort (p=0.012, **Figure 7E**). Nevertheless, when further stratifying the patients according to immunotherapeutic response types, the progressive disease, stable disease and partial response groups showed different risk scores (**Figure 7F**). We also grouped the therapeutic response in a binary mode, and found that the complete/partial response group had a higher percentage of high scores than the stable/progressive disease group (**Figure 7G**). Besides, glioma patients with high risk score were less likely to benefit from chemotherapy or radiotherapy (**Figures 7H, I**, respectively).

## Discussion

In the present study, monocyte density was explored as a marker for glioma prognosis by using WGCNA for the first time. Genes derived from the module of WGCNA were used for glioma patient grouping. Machine learning including neural network and SVM were applied for validating the clustering results based on monocyte. An extensive annotation of tumor genomics, TME, clinical traits, metabolism, and hypoxia was performed for monocyte-related patient groups. A risk score based on the DEGs between monocyte -related clusters was generated by PCA, with biological functions, immune subtypes, and immunotherapeutic response associated with the risk score being explored.

As a major population of innate immune cells, monocyte exert its two-sided roles in facilitating tumor growth (24) and inhibiting metastatic spread of tumor (25). Notably, monocytes could primarily differentiate into tumorigenic TAMs, yet monocytes could also differentiate into DCs responsible for effective adaptive immune responses. TAMs are recruited early during tumor formation of GBM and contribute to tumor development (26). Dendritic Cell-based Vaccination has been proved with potential therapeutic effect on GBM patient survival (27). Moreover, monocytes closely adhere to GBM via *VCAM-1* and promotes tumor invasion activity (10). In summary, monocytes could both directly or indirectly mediate the tumor growth and invasion of gliomas.

To explore and confirm the predictive value of monocyte in gliomas, monocyte entity is defined by the consensus-based xCell algorithm in the present study. The WGCNA-derived monocyte-related genes clustered glioma patients into two groups with distinctive clinical traits and immune characteristics. It should be noted that even though almost all GBM patients fell in cluster 1, some LGG patients also fell in cluster 1. Therefore, the clustering results are more likely to reflect the similar molecular characteristics of GBM and LGG. Patients in Cluster 1, with worse survival, had higher level of *IDH* WT, 1p19q noncodeletion, and *MGMT* promoter unmethylation that all correlated with a more malignant phenotype of glioma. Further, patients in cluster 1 was more associated with hypoxia and hypermetabolism, both of which correlated with the malignancy of cancer. Immune infiltrating cells such as fibroblasts, M2 macrophages, and DCs had higher expression in Cluster 1. Classical immune checkpoint molecules such as *PD1*, *PDCD1LG2*, *LAG3*, and *VTCN1* all had higher expression in cluster 1. Additionally, patients in cluster 1 had higher ESTIMATE scores. Taken together, monocyte served as an effective factor stratifying glioma patients with diverse clinical features and outcomes.

The genomic alteration related to monocyte entity was then investigated. The *IDH* missense mutations confer better survival outcome in glioma patients. Nevertheless, LGGs carrying the *IDH* mutations are more prone to develop into secondary GBMs, especially when tertiary genetic alterations in oncogenes like *PIK3CA* and *PDGFRA* occur in the same patient (28). The present study finds that the *IDH1* missense mutations are overrepresented in the cluster 2 (92%) compared with the cluster 1 (12%), in accordance with previous findings that *IDH* mutations are more enriched in LGGs than in high grade ones (29). Likewise, *EGFR*, which is the most enriched mutated gene in cluster 1 (28%) as identified by somatic mutation analysis, has been reported to be frequently activated in GBM (30).

Based on the DEGs identified between two clusters, a risk score calculated by 33 monocyte related genes was reached. Monocyte related risk score showed high efficiency in predicting patients’ 3 years and 5 years survival probability. A nomogram incorporating monocyte further confirmed the efficacy of monocyte as a prognostic marker.

In recent years, acumulating evidence proved that the tumor immune microenvironment played an important role in cancer development (31). Central nervous system was long considered as an “immune privileged” organ due to the existence of blood brain barrier. Currently, the discovery of lymphatic vessels has subverted this opinion, in which immune cells could infiltrate into the brain during tumor progression as a part of the tumor immune microenvironment (32). We next tried to establish a robust relationship between risk score and tumor immune microenvironment. Glioma cells secret *CCL-2* to promote the activity of tumor-associated macrophages which suppressed the activities of cytotoxic T cells (33). Besides, glioma cells increase the expression of programmed cell death 1 ligand (*PD-L1*), a classical immune checkpoint molecule, which induces the immunosuppressive context and mediates the immune escape of tumor cells (34). High risk score group tended to correlate with more immune infiltrating cells, such as macrophages and fibroblasts. High risk score group also expressed more immune checkpoint molecules including *PDCD1* and chemokines *CCL-5*, *CXCL10*, and *CXCL9*. Functional annotation of risk score further revealed that macrophage activation, fibroblast proliferation, and regulation of T cell apoptotic process were more frequently occurred in glioma patients with high risk score. A series of factors associated with tumor immunogenicity and antigen presentation capacity such as MSI, intratumor heterogeneity, and neoantigens were found to be highly expressed in high risk score group. MSI has been recently reported to predict patients’ responses to immunotherapy, and has been proposed as a promising biomarker for anti-PD-L1 therapy (35). Likewise, intratumor heterogeneity has also been proved to influence the outcome of immunotherapy (36). It is noteworthy that therapeutic approaches based on neoantigens, another biomarker in cancer immunotherapy, have been proposed to selectively enhance T cell reactivity (22). Besides, *IFN-γ* Dominant and Lymphocyte Depleted immune subtypes were more observed in high risk score group. The above findings indicated that risk score was associated with an immunosuppressive and tumorigenic microenvironment.

Immunotherapy, represented by anti-*PD-1* therapy, has been regarded as a promising therapeutic option in melanoma and urothelial cancer (37–41). So far, clinical trials of immunotherapy have not demonstrated satisfactory results in glioma patients. Based on the IMvigor210 cohort, our analyses showed that the high risk score group had more frequent stable/progressive disease patients than responsive patients, representing a worse response to immunotherapies. Based on the CGGA693 cohort, high risk score group receiving chemotherapy or radiotherapy was associated with worse survival. Therefore, we hypothesized that monocyte could be an effective factor in predicting glioma patients’ response to immunotherapy and classical chemoradiotherapy.

In conclusion, our analyses identified a monocyte gene signature consisting of 33 monocyte-specific genes, and established its prognostic value in glioma. Our findings strongly supported a modulatory role of monocytes in glioma progression and proved that monocyte served as an effective factor stratifying glioma patients’ survival probability. One major limitation of this study was the lack of external real-world data to confirm and support our findings. Another limitation was the lack of GBM cohort for predicting the immunotherapy response, which the tumor microenvironment in urothelial carcinoma might be different from that in GBM. Thus, a GBM cohort was expected to validate the efficacy of monocyte-derived risk score in predicting immunotherapy response in the future. Moreover, the in-depth mechanisms such as governing the differentiation of monocytes into protumoral or antitumoral cells in TME of gliomas remained undermined and needed further experiment for validation. Additionally, the underlying regulatory role of monocyte in immune responses remained to be elucidated.

## Data Availability Statement

All data used in this work can be acquired from the Cancer Genome Atlas (TCGA) datasets (https://xenabrowser.net/), the Chinese Glioma Genome Atlas (CGGA) datasets (http://www.cgga.org.cn/), and GSE108474 from the GEO database (https://www.ncbi.nlm.nih.gov/geo/).

## Author Contributions

HZ, QC, WW, NZ, YZ, HC, and ZD designed and drafted the manuscript. HZ wrote figure legends and revised the manuscript. QC, HZ, and NZ conducted data analysis. All authors contributed to the article and approved the submitted version.

## Funding

Financial support was provided by the National Natural Science Foundation of China (NO. 82073893), China Postdoctoral Science Foundation (NO. 2018M633002), Hunan Provincial Natural Science Foundation of China (NO.2018JJ3838), Hunan Provincial Health Committee Foundation of China (C2019186). Xiangya Hospital Central South University postdoctoral foundation.

## Conflict of Interest

The authors declare that the research was conducted in the absence of any commercial or financial relationships that could be construed as a potential conflict of interest.

## Abbreviations

CDF, cumulative distribution function; CGGA, Chinese Glioma Genome Atlas; CNA, copy number alternations; CNV, copy number variation; GBM, glioblastoma; GEO, Gene Expression Omnibus; GO, gene ontology; GSEA, gene set enrichment analysis; GSVA, gene set variation analysis; KEGG, Kyoto Encyclopaedia of Genes and Genomes; LGG, low grade glioma; PCA, principal component analysis; TGF-β, tumor growth factor-β; SNP, single-nucleotide polymorphism; SNV, single-nucleotide variant; TAM, tumor associated macrophage; TCGA, The Cancer Genome Atlas; TME, tumor microenvironment; WGCNA, weighted gene co-expression network analysis; PD-L1, programmed cell death 1 ligand; DEG, differentially expressed genes; VCAM-1, vascular cell adhesion molecule-1; DC, dendritic cell; PAM, partition around medoids; MSI, microsatellite instability; HRD, homologous recombination deficiency; CTA, cancer testis antigen.
