# Supplementary material for: The Predictive Value of Monocytes in Immune Microenvironment and Prognosis of Glioma Patients Based on Machine Learning
Source: Front Immunol. 2021 Apr 16;12:656541. doi: 10.3389/fimmu.2021.656541 (PMC8095378; doi:10.3389/fimmu.2021.656541)
Supplement: Supplementary file 1 [file DataSheet_1.zip › Supplementary Figures.docx]

Figure Legends:


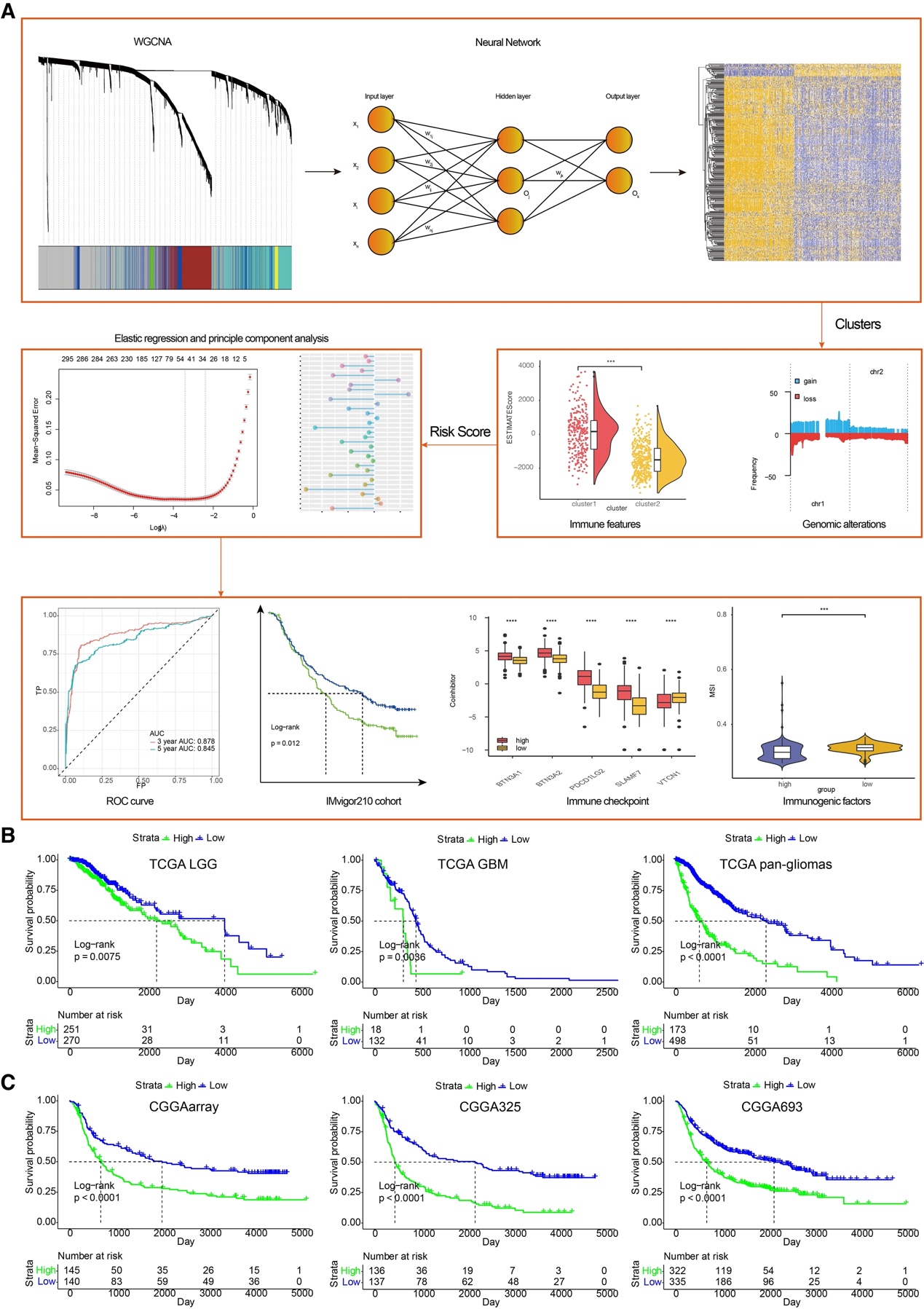


Figure S1. A. Flow chart of the present study. B. Stratification of samples by low and high monocyte levels and the subsequent survival analysis in LGG, GBM, and pan-gliomas from TCGA. C. Stratification of samples by low and high monocyte levels and the subsequent survival analysis in CGGAarray, CGGA325, and CGGA693.


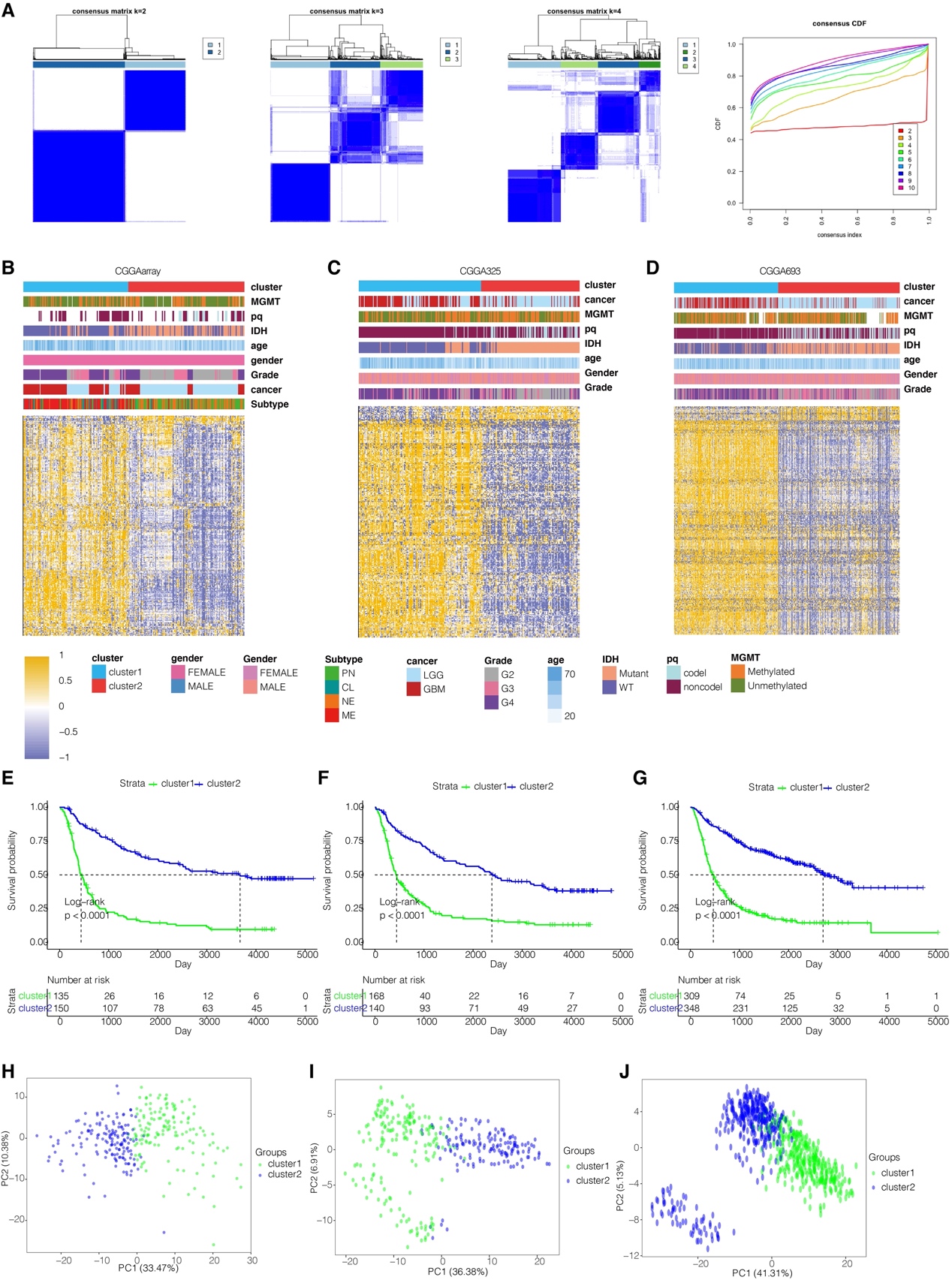


Figure S2. A. Clustering stability analysis by ConsensusClusterPlus package. Using data derived from the CGGAarray (B), CGGA325 (C) and CGGA693 (D) datasets, heatmap plots showed that samples from clusters 1 and 2 had different demographical features, tumor pathologies and mutation status. E. Survival analysis of the two clusters in the CGGA array dataset. F. Survival analysis of the two clusters in the CGGA325 dataset. G. Survival analysis of the two clusters in the CGGA693 dataset. H. Sample clustering by PCA in the CGGA array dataset. I. Sample clustering by PCA in the CGGA325 dataset J. Sample clustering by PCA in the CGGA693 dataset.


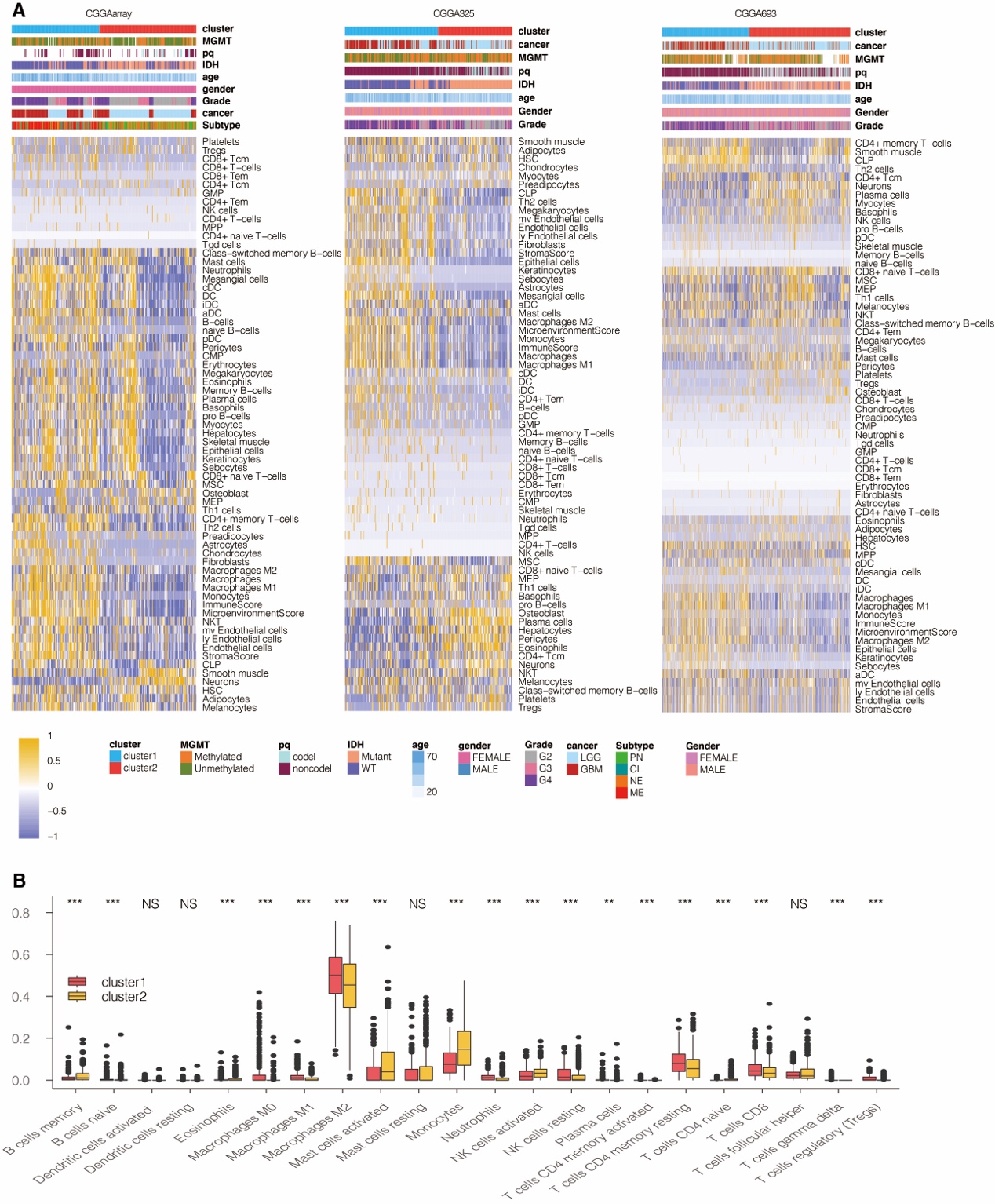


Figure S3. A. Dendrogram correlating the levels of 64 cell types and clusters in CGGAarray, CGGA325, and CGGA693. B. Expression level of 22 immune cell types in two clusters in TCGA.


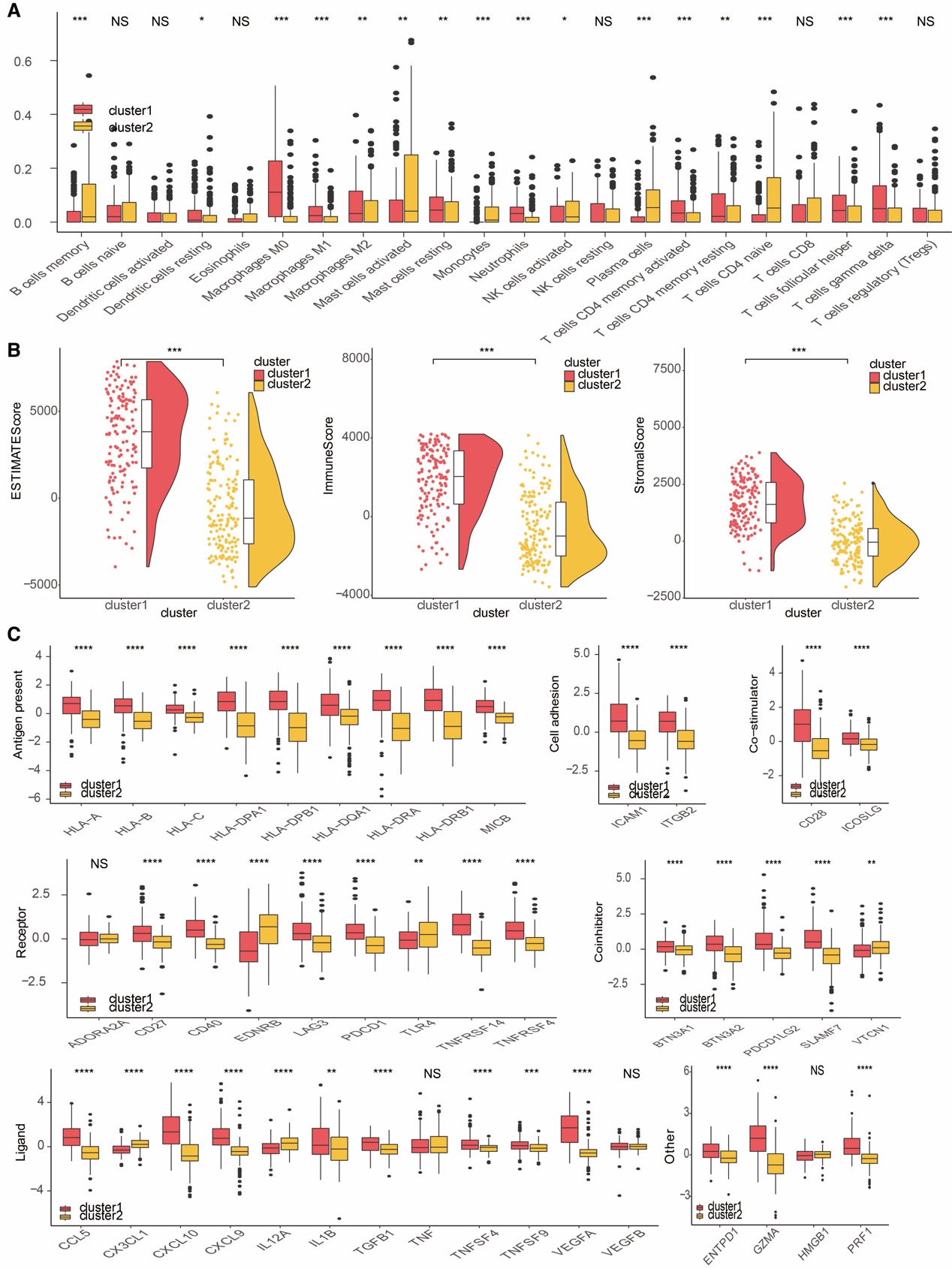


Figure S4. A. Expression level of 22 immune cell types in two clusters in CGGAarray. B. ESTIMATEScores, ImmuneScores and StromalScores of the two clusters in CGGAarray. C. Molecule levels of immune checkpoints in two clusters in CGGAarray.


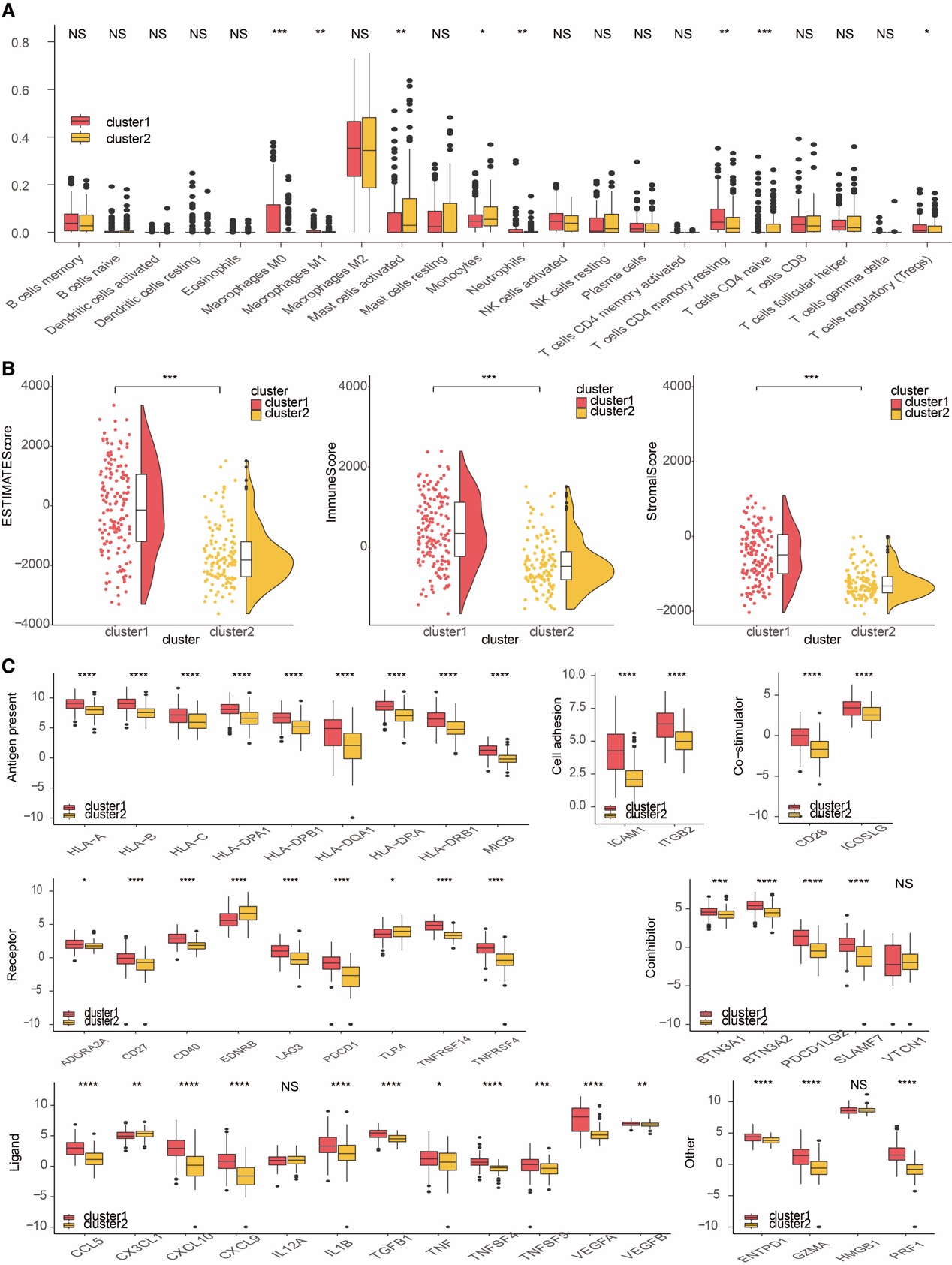


Figure S5. A. Expression level of 22 immune cell types in two clusters in CGGA325. B. ESTIMATEScores, ImmuneScores and StromalScores of the two clusters in CGGA325. C. Molecule levels of immune checkpoints in two clusters in CGGA325.


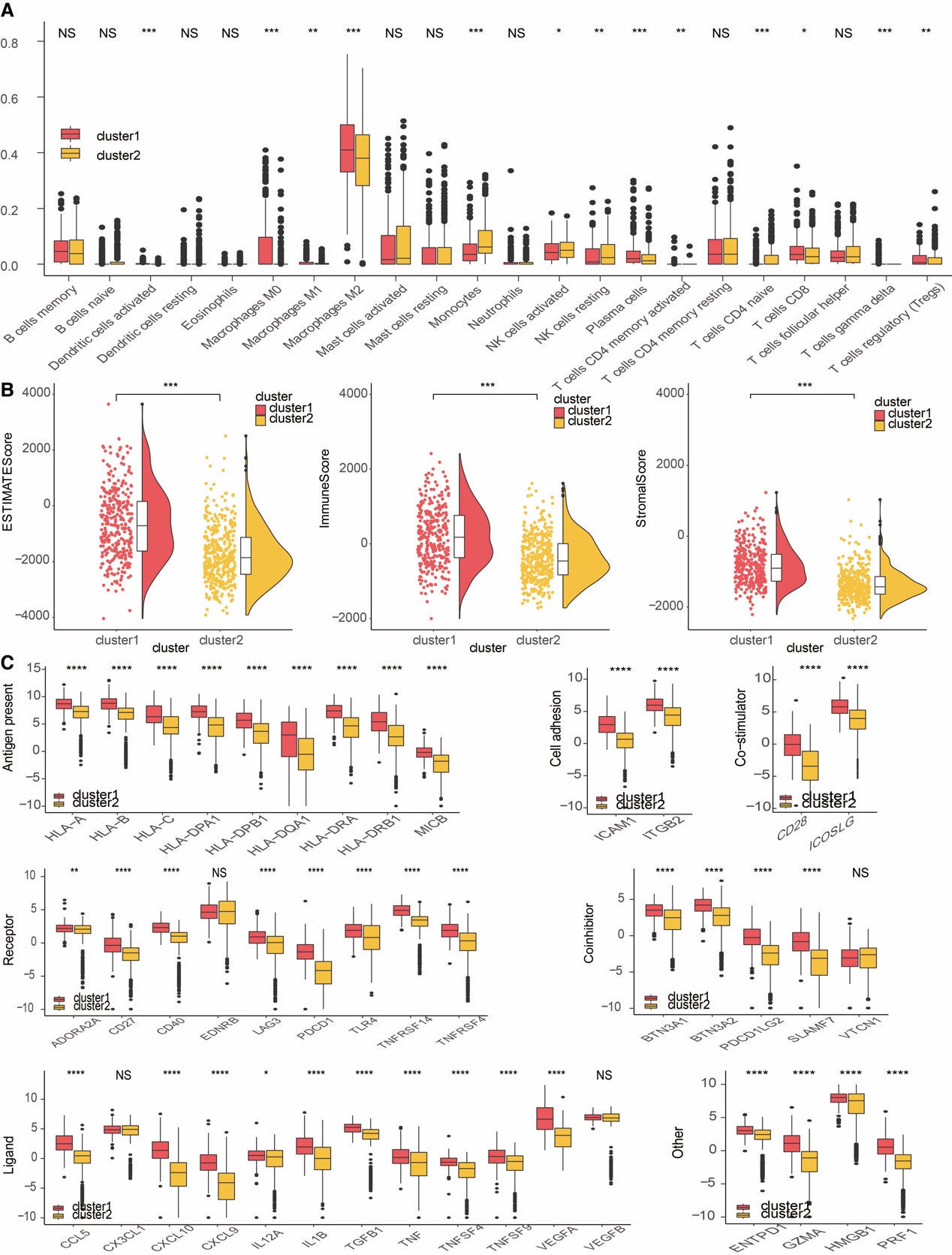


Figure S6. A. Expression level of 22 immune cell types in two clusters in CGGA693. B. ESTIMATEScores, ImmuneScores and StromalScores of the two clusters in CGGA693. C. Molecule levels of immune checkpoints in two clusters in CGGA693.


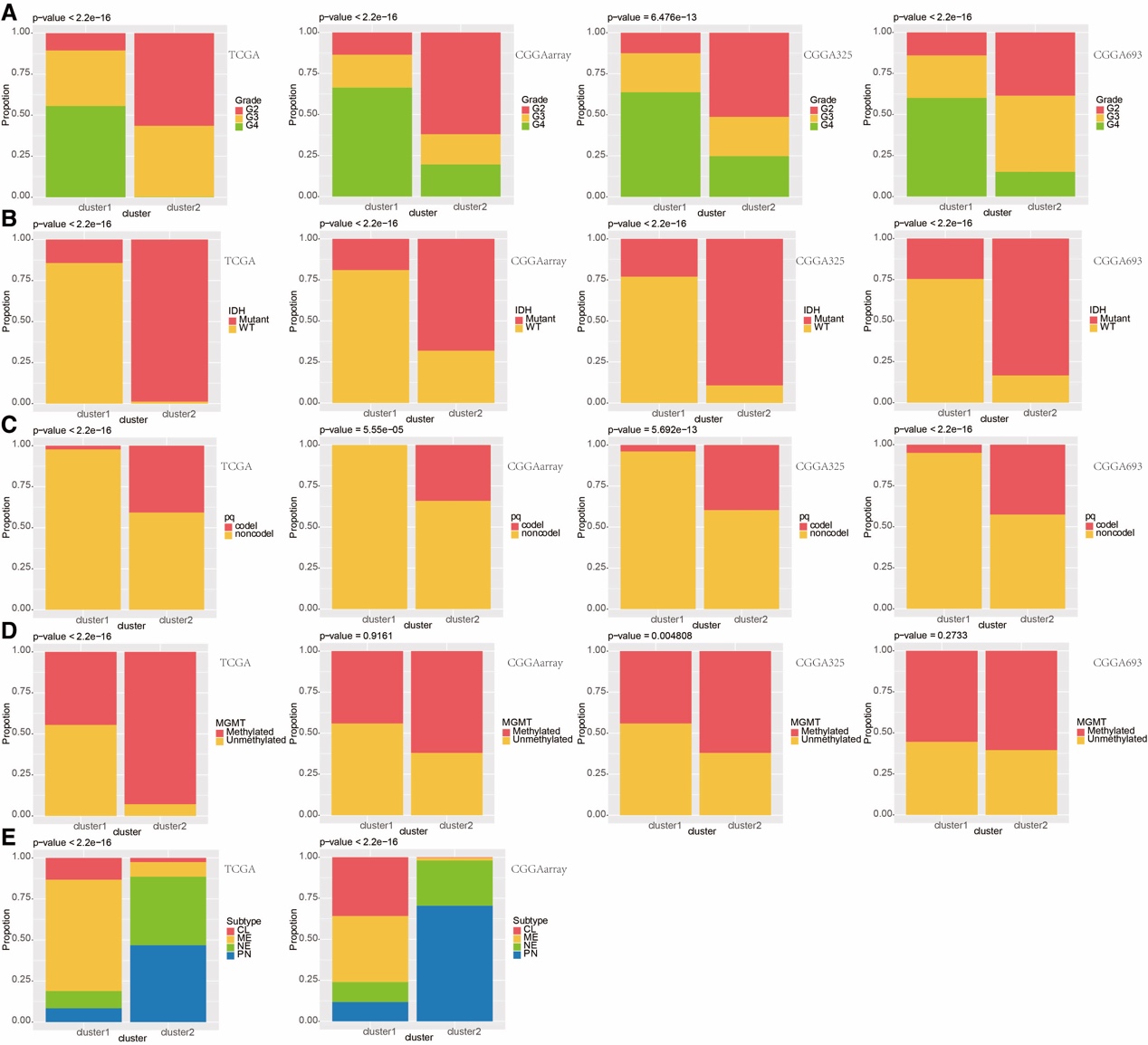


Figure S7. A. The proportions of different tumor grades in TCGA, CGGAarray, CGGA325, and CGGA693. B. Samples with or without the *IDH* mutation in TCGA, CGGAarray, CGGA325, and CGGA693. C. Samples with or without the chromosome 1p/19q codeletion in TCGA, CGGAarray, CGGA325, and CGGA693. D. Samples with or without the *MGMT* promoter methylation in TCGA, CGGAarray, CGGA325, and CGGA693. E. The four GBM subtypes in the two clusters in TCGA and CGGAarray.


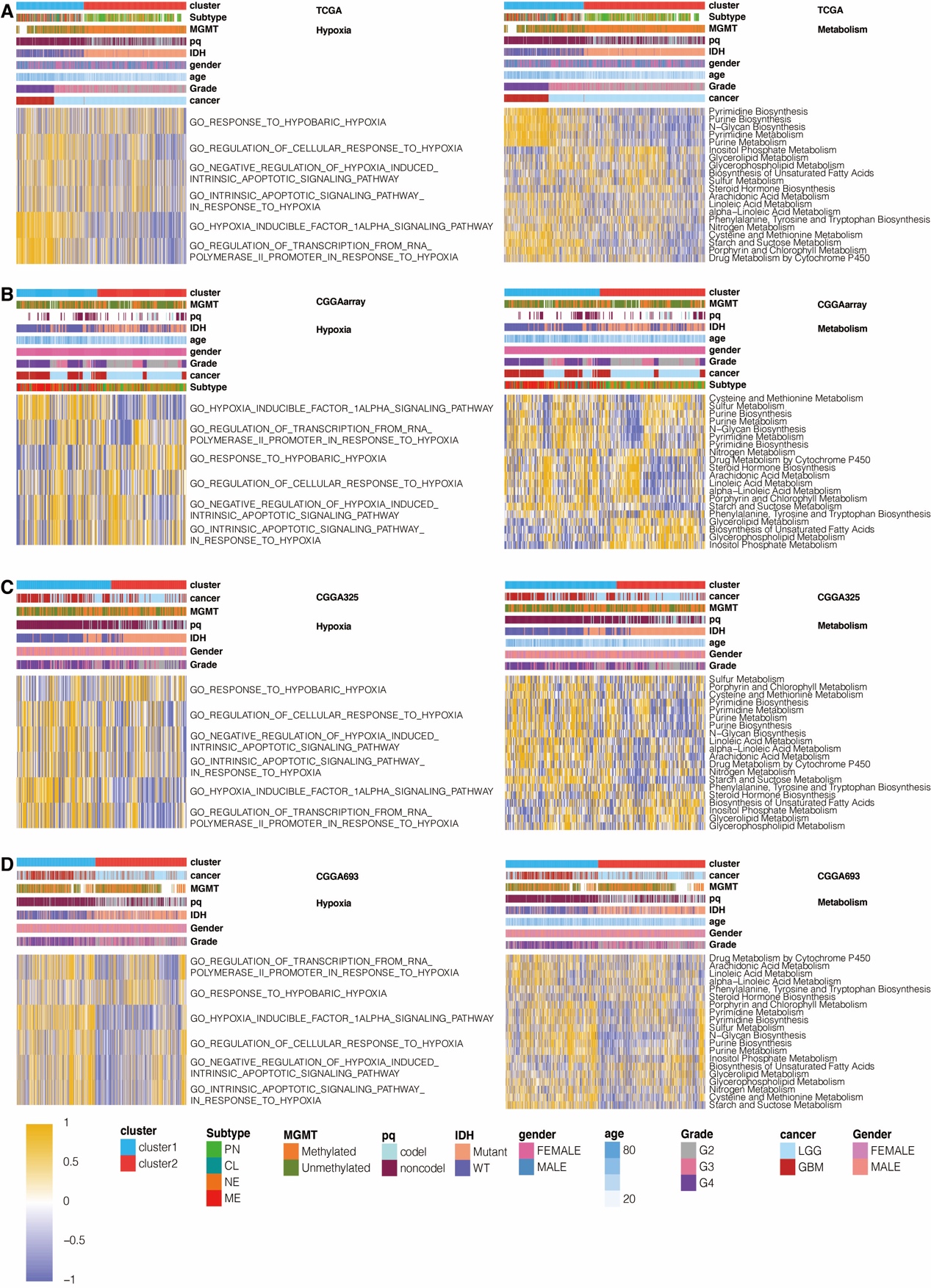


Figure S8. A. GO functional enrichment analysis of hypoxia and metabolism in the two clusters in TCGA. B. GO functional enrichment analysis of hypoxia and metabolism in the two clusters in CGGAarray. C. GO functional enrichment analysis of hypoxia and metabolism in the two clusters in CGGA325. D. GO functional enrichment analysis of hypoxia and metabolism in the two clusters in CGGA693.


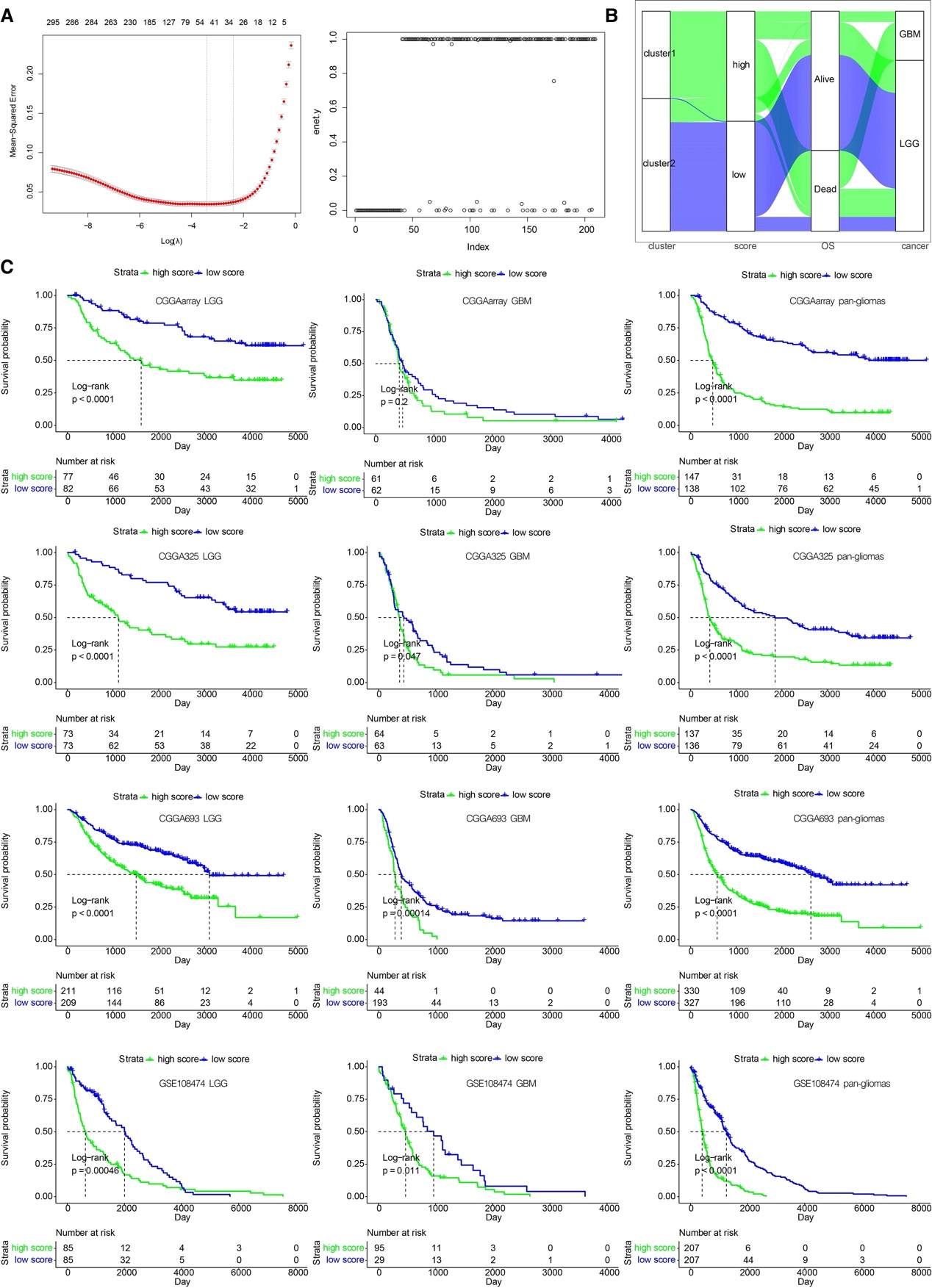


Figure S9. A. identification of 33 monocyte-related genes by elastic net regression analysis and PCA algorithm. B. Sankey diagram visualizing the correlation of clusters, risk score, overall survival and cancer types. C. Survival analyses of risk scores in pan-glioma, LGG and GBM groups from CGGAarray, CGGA325, CGGA693, and GSE108474 datasets.


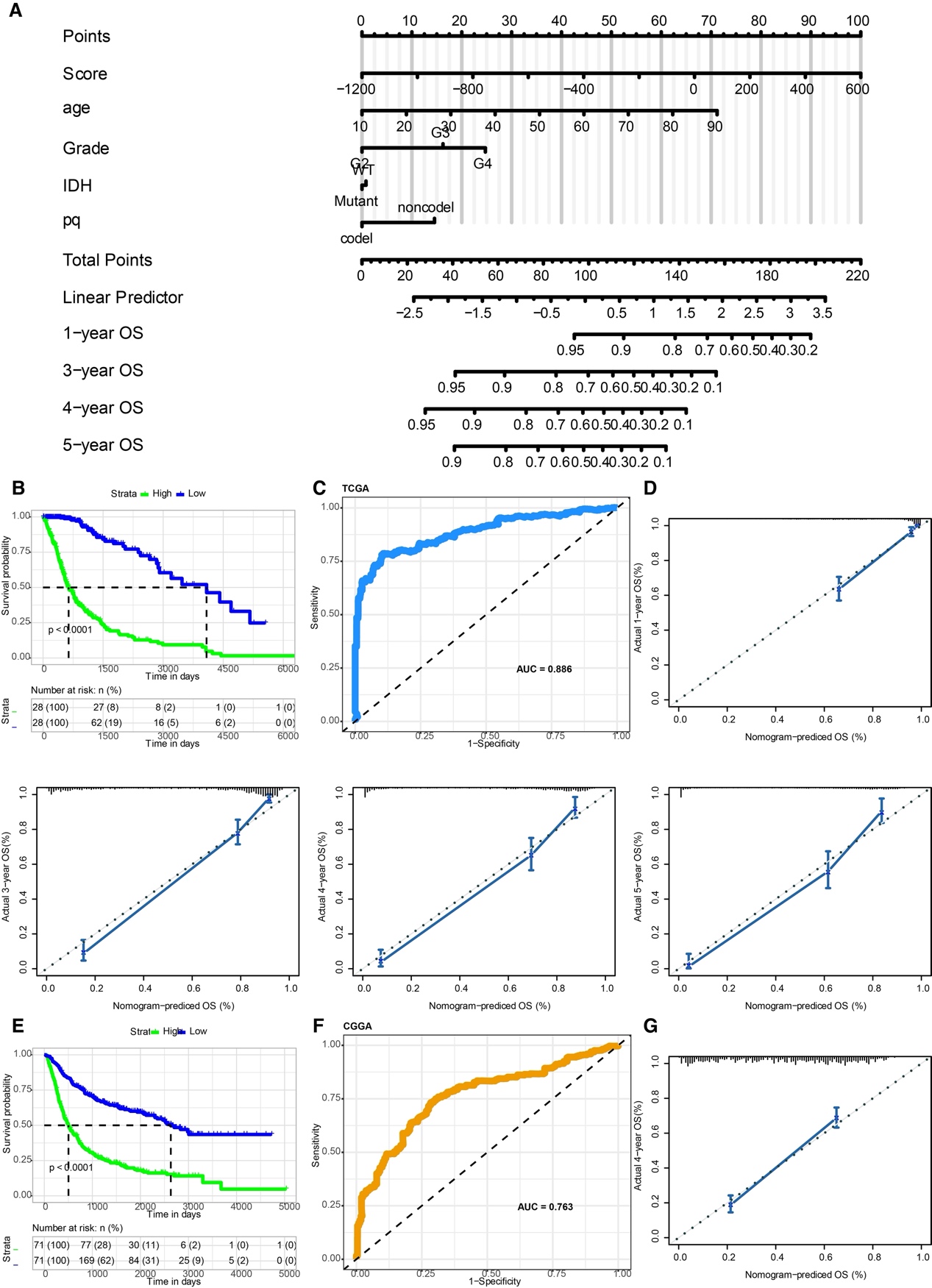


Figure S10. A. A nomogram integrating M2 scores, clinical, genetic, pathological variables and overall survival probability. B. Survival analysis of the two clusters in TCGA dataset. C. ROC curve plot of evaluating the nomogram in TCGA dataset. D. Correlation of actual and predicted overall survival rates in TCGA dataset. E. Survival analysis of the two clusters in CGGA693 dataset. F. ROC curve plot of evaluating the nomogram in CGGA693 dataset. G. Correlation of actual and predicted overall four-year survival rates in CGGA693 dataset.


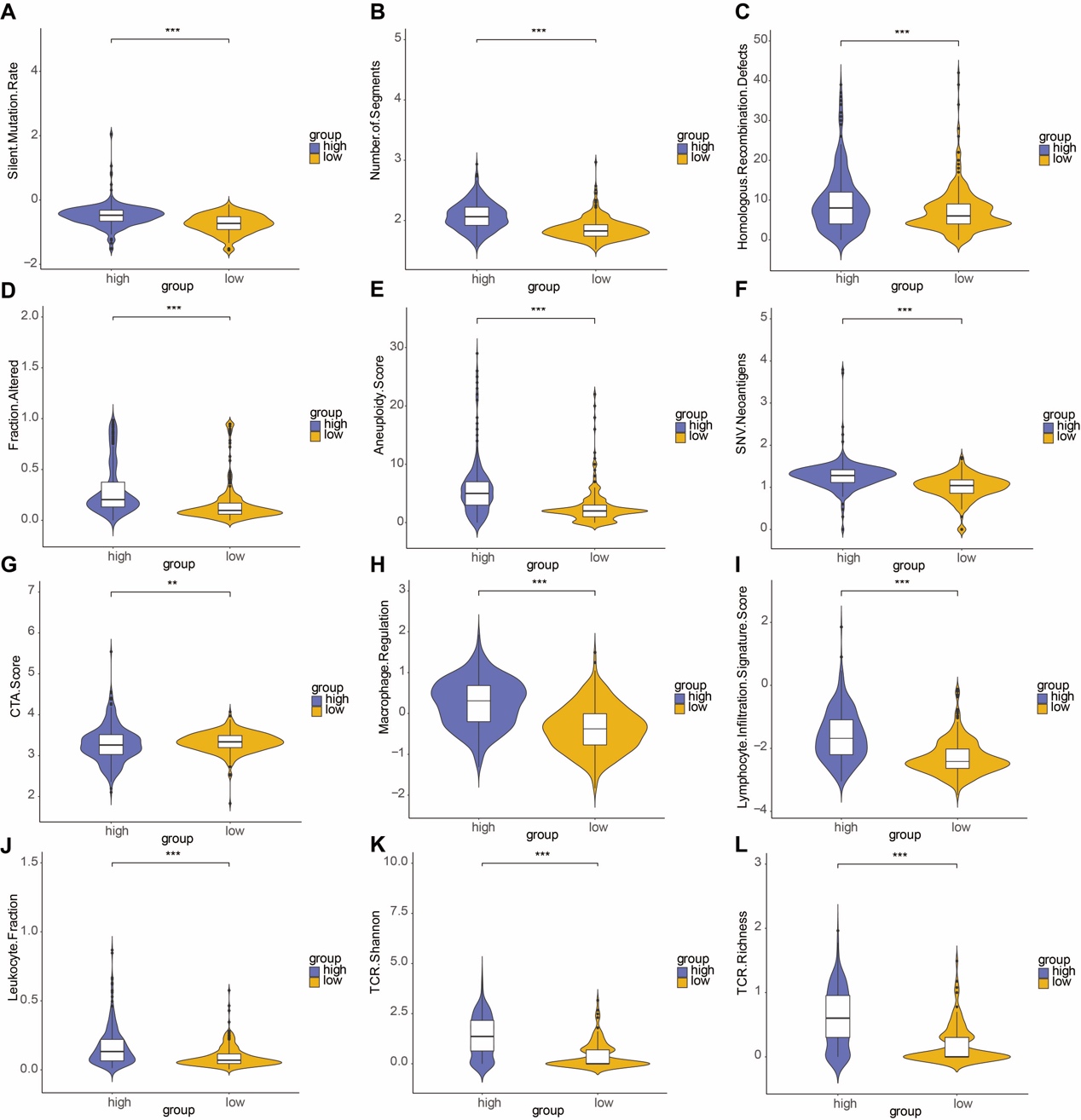


Figure S11. Immunogenic features of risk scores. A. Silent Mutation Rate in high and low risk score. B. Number of Segments in high and low risk score. C. Homologous Recombination Defects in high and low risk score. D. Fraction Altered in high and low risk score. E. Aneuploidy Score in high and low risk score. F. SNV Neoantigens in high and low risk score. G. CTA Score in high and low risk score. H. Macrophage Regulation in high and low risk score. I. Lymphocyte Infiltration Signature Score in high and low risk score. J. Leukocyte Fraction in high and low risk score. K. TCR Shannon in high and low risk score. L. TCR Richness in high and low risk score.

**Supplementary tables**

Table S1 All CNV regions compared between two clusters

Table S2 Significantly different CNV regions compared between two clusters
